# Resistance Analyses of Integrase Strand Transfer Inhibitors within Phase 3 Clinical Trials of Treatment-Naive Patients

**DOI:** 10.3390/v6072858

**Published:** 2014-07-22

**Authors:** Kirsten L. White, Francois Raffi, Michael D. Miller

**Affiliations:** 1 Department of Clinical Virology, Gilead Sciences, Inc., 333 Lakeside Drive, Foster City, CA 94404, USA; E-Mail: michael.miller@gilead.com; 2 Department of Infectious Diseases, University Hospital, Hotel-Dieu, 1 Place Ricordeau, Nantes 44093, France; E-Mail: francois.raffi@wanadoo.fr

**Keywords:** HIV, drug resistance, integrase, clinical trial

## Abstract

The integrase (IN) strand transfer inhibitors (INSTIs), raltegravir (RAL), elvitegravir (EVG) and dolutegravir (DTG), comprise the newest drug class approved for the treatment of HIV-1 infection, which joins the existing classes of reverse transcriptase, protease and binding/entry inhibitors. The efficacy of first-line regimens has attained remarkably high levels, reaching undetectable viral loads in 90% of patients by Week 48; however, there remain patients who require a change in regimen due to adverse events, virologic failure with emergent resistance or other issues of patient management. Large, randomized clinical trials conducted in antiretroviral treatment-naive individuals are required for drug approval in this population in the US, EU and other countries, with the primary endpoint for virologic success at Week 48. However, there are differences in the definition of virologic failure and the evaluation of drug resistance among the trials. This review focuses on the methodology and tabulation of resistance to INSTIs in phase 3 clinical trials of first-line regimens and discusses case studies of resistance.

## 1. Introduction

The treatment of many patients infected with HIV-1 has become relatively straightforward for first-line regimens based on international treatment guidelines. The most recent class of HIV-1 inhibitors to be approved target the HIV-1 integrase (IN) gene via the inhibition of the strand transfer step: IN strand transfer inhibitors (INSTIs) [1]. This new class of INSTIs consists of raltegravir (RAL), elvitegravir (EVG) and dolutegravir (DTG). A recent systemic review and meta-analysis of clinical studies of INSTIs supports the use of INSTIs in first-line regimens [2].

Approval of an HIV drug in treatment-naive individuals requires the rigorous testing of the drug alone and in combination with other inhibitors through the clinical development process across phase 1, 2 and 3 studies. The final phase of the drug development process prior to submission for review and approval by regulatory bodies are the large, randomized, double-blind, phase 3 studies [3,4]. In this review, the major phase 3 clinical trials of INSTIs designed by their sponsoring pharmaceutical companies are summarized. The subtle differences in the analysis of resistance are discussed using a set of case studies spanning a range of virologic success and failure profiles. 

### 1.1. Treatment Guidelines for the HIV-1-Infected Antiretroviral-Naive Patient

Major guidelines for the treatment of HIV-1-infected patients provide recommendations for when to treat, what to treat with and how to monitor for virologic failure. The most influential guidelines are the United States Department of Health and Human Services (DHHS), International Antiviral (formerly AIDS) Society-United States of America (IAS-USA), European AIDS Clinical Society (EACS), World Health Organization (WHO) and the British HIV Association (BHIVA) [5,6,7,8,9,10]. These guidelines are not entirely consistent with one another on all points. They are also updated at different intervals as new information arises. The DHHS and IAS-USA guidelines recommend the initiation of treatment for all HIV-1-infected patients [7,8]. Other current guidelines recommend treatment of HIV-1 infection in all patients with CD4 <350 cells/μL and with higher CD4 counts in patients with other risk factors, such as hepatitis co-infections, pregnancy or older age, or to prevent HIV-1 transmission. 

The guidelines also differ in their choice of “recommended” antiretrovira**l** regimens and not all have been updated to include the INSTI class in first-line therapy. All guidelines recommend a nucleos(t)ide reverse transcriptase inhibitor (NRTI) backbone typically consisting of a fixed-dose combination of either emtricitabine and tenofovir disoproxil fumarate (FTC/TDF) or abacavir and lamivudine (ABC/3TC). For the third agent, other antiretroviral classes, including non-nucleoside reverse transcriptase inhibitors (NNRTIs) or ritonavir-boosted protease inhibitors (PIs), are recommended, but here, we will focus on the INSTI class. For the use of INSTIs as the first line, the DHHS includes all three INSTIs as recommended first line regimens: RAL + FTC/TDF, EVG/COBI/FTC/TDF (the single tablet regimen of cobicistat (COBI)-boosted EVG with FTC/TDF), DTG + ABC/3TC and DTG + FTC/TDF [8,9]. The IAS-USA guidance for INSTIs, last updated in 2012, recommends RAL + FTC/TDF and lists EVG/COBI/FTC/TDF or RAL + ABC/3TC as alternative regimens [7]. The EACS guidance recommends RAL + either ABC/3TC or FTC/TDF and categorizes EVG/COBI/FTC/TDF as an alternative regimen [6]. The BHIVA guidance recommends RAL or EVG/COBI with two NRTIs consisting of FTC/TDF or, if the viral load is <100,000 copies/mL, ABC/3TC [5]. The WHO guidance recommends INSTIs in third-line regimens [10].

The definition of virologic failure and when to change to a second-line regimen also vary between guidelines. The DHHS guidelines define virologic failure and recommend resistance testing for visits with HIV-1 RNA >1000 copies/mL (after having achieved virologic suppression) and suggest considering resistance testing at viral loads between 500 and 1000 copies/mL [8,9]. The BHIVA guidance defines virologic failure as a visit with HIV-1 RNA >400 copies/mL and suggests a repeat viral load test to confirm the failure [5]. The IAS-USA guideline defines virologic failure as HIV-1 RNA >50 copies/mL after 24 weeks of treatment [7]. EACS has similar criteria as IAS-USA and suggests resistance testing for two consecutive visits with a viral load >50 copies/mL, especially for viral loads above 350 copies/mL [6]. Clearly, there is not a consistent recommendation for the definition of virologic failure and when to conduct resistance testing. This lack of standardization is also reflected in phase 3 clinical trials.

### 1.2. INSTI Resistance

The INSTIs have potent antiviral activity. All currently approved INSTIs are small molecules that share key chemical motifs and bind to the active site of IN [1,11,12]. The INSTIs have low nanomolar effective concentration required to reach 50% virus inhibition (EC50) values against HIV-1 and HIV-2. The INSTIs RAL and EVG have largely similar resistance profiles. The primary resistance mutations reside in the active site of IN. Resistance to RAL involves three major pathways containing N155H, Y143C/H/R or Q148H/K/R [13]. Major resistance pathways to EVG include T66I, E92Q, N155H and Q148H/K/R [14].* In vitro* resistance selections found that primary mutations can emerge rapidly in tissue culture.* In vivo*, INSTI resistance can also rapidly occur in patients treated with RAL or EVG when not supported by a fully-active, two-drug backbone [15,16]. The most recent INSTI approved was DTG, which has a higher resistance barrier than the two other INSTIs. This has been attributed to an extended linker region that allows farther entry into the IN active site pocket to make more contacts with the invariant viral DNA and an increased flexibility that allows DTG to readjust its position to better tolerate single INSTI resistance mutations [17]. 

## 2. Clinical Trials of INSTI-Based First-Line Regimens

### 2.1. Resistance Exclusion Criteria in Clinical Studies

In developed countries, it is standard to conduct HIV-1 genotyping prior to starting the first antiretroviral regimen. This is important because an estimated 10%–15% of treatment-naive patients harbor primary drug resistance mutations in the PR and RT coding regions of the pol gene [18]. Because INSTIs were relatively recently introduced, transmitted INSTI resistance (-R) is rare, and only three cases have been documented in the literature [19,20,21]. There are, however, some INSTI-R mutations, such as T97A, that are rare, polymorphic mutations [22,23]. These polymorphic mutations also vary by HIV-1 subtype [24,25]. In the seven phase 3 trials reviewed here, no genotyping of the IN coding region was conducted as part of the screening or entry requirements. As the potential for transmitted INSTI-R increases, standard IN genotyping may be implemented in regions with higher INSTI use and if transmitted INSTI-R becomes more frequent. 

### 2.2. Viral Load Tests

Clinical trials of HIV-1 utilize the plasma HIV-1 RNA concentration, referred to as the viral load, as the primary biomarker of antiviral drug efficacy. International guidelines recommend targeting an undetectable viral load. In the first decade of clinical trials of HIV-1, the viral load target was set to achieving <400 copies/mL, which was the lower limit of detection of the assays available at the time. The lower limit of detection for viral load assays has been subsequently improved. A viral load detection limit of <75 copies/mL was achieved using the branched DNA (bDNA) assay (currently, the Versant^®^ HIV-1 RNA 3.0 assay (Siemens Medical Solutions Diagnostics)), and <50 copies/mL using the COBAS Amplicor HIV-1 Monitor Test, version 1.5 (Roche Diagnostics). Presently, real-time PCR approaches have further lowered the limit of detection to 40 copies/mL using the Abbott RealTime HIV-1 PCR assay [26] and 20 copies/mL using the Roche COBAS TaqMan HIV-1 Test version 2.0 assay [27]. Most registrational clinical trials, however, use a target of <50 copies/mL as their primary endpoint. Other viral load targets are utilized by the AIDS Clinical Trials Group (ACTG), where <200 copies/mL is the standard endpoint [28]. Most INSTI trials have used the Amplicor v1.5 assay, but this test is now discontinued. Therefore, future clinical trials will be using other assays, such as RealTime or TaqMan v2.0. Of the studies reviewed here, the STARTMRK, QDMRK, GS-US-236-0102 and GS-US-236-0103 studies measured HIV-1 RNA concentration using the standard and ultrasensitive COBAS Amplicor HIV-1 Monitor assay (v1.5) [29,30,31,32]. The SPRING-2, SINGLE and FLAMINGO studies measured HIV-1 RNA concentration using the RealTime HIV-1 PCR assay (Abbott Molecular) [33,34,35]. All seven trials used a centralized lab for their HIV-1 viral load testing.

### 2.3. Primary Efficacy Outcomes in INSTI Trials

The study designs and Week 48 efficacy outcomes for the seven trials of INSTI-based therapy reviewed here are shown in Table 1.

Two studies evaluated RAL as the first-line therapy. STARTMRK was an international, double-blind, phase 3 randomized trial comparing 400 mg RAL twice a day plus FTC/TDF* vs*. efavirenz (EFV) plus FTC/TDF [29]. At Week 48, in the per protocol, non-completer equals failure analysis, 86% (*n =* 241/280) of the RAL group achieved the primary endpoint of <50 copies/mL of HIV-1 RNA at Week 48, compared with 82% (*n =* 230/281) of the EFV group (difference 4.2%, 95% CI −1.9% to 10.3%), indicating that RAL was non-inferior to EFV (*p <* 0.0001 for non-inferiority) [29]. The QDMRK study (MK-0518 protocol 071) compared 800 mg of RAL taken once daily (QD) or 400 mg of RAL taken twice daily (BID), both in combination with FTC/TDF in an international, double-blind, phase 3, randomized trial [30]. The primary endpoint at Week 48, by intention-to-treat, non-completer equals failure analysis, found that 83% (*n* = 318/382) in the QD group compared with 89% (*n* = 343/386) in the BID group had HIV-1 RNA <50 copies/mL (difference −5.7%, 95% CI −10.7 to −0.83; *p* = 0.044). The once-daily dosing was to be regarded as non-inferior to twice-daily dosing if the lower bound of the 95% CI for the difference was above −10%; therefore, the reported, one-sided *p* = 0.44 failed to show non-inferiority. Based on this data, the once-daily RAL dose was not recommended. 

**Table 1:** Phase 3 clinical trials of integrase strand transfer inhibitor (INSTI)-based regimens as first-line therapy.

INSTI	Clinical Trial	Treatment Groups (n)	Week 48 Treatment Outcome (HIV-1 RNA <50 Copies/mL)	Interpretation	Reference
RAL	STARTMRK	RAL (BID) + FTC/TDF (*n =* 281)* vs*. EFV + FTC/TDF (*n =* 282)	86.1% *vs*. 81.9% (difference 4.2%, 95% CI −1.9 to 10.3)	Non-inferiority	[29]
	QDMRK	RAL (QD) + FTC/TDF (*n =* 382)* vs*. RAL (BID) + FTC/TDF (*n =* 388)	83.2% *vs*. 88.9% (difference −5.7%, 95% CI −10.7 to −0.83)	Not non-inferiority	[30]
EVG	GS-US-236-0102	EVG/COBI/FTC/TDF (*n =* 348)* vs*. EFV/FTC/TDF (*n =* 352)	87.6% *vs*. 84.1% (difference 3.6%, 95% CI –1.6 to 8.8)	Non-inferiority	[31]
	GS-US-236-0103	EVG/COBI/FTC/TDF (*n =* 353)* vs*. ATV + RTV + FTC/TDF (*n =* 355)	89.5% *vs*. 86.8% (difference 3.0%, 95% CI –1.9 to 7.8).	Non-inferiority	[32]
DTG	SPRING-2	DTG + [FTC/TDF or ABC/3TC] (*n =* 411)* vs*. RAL + [FTC/TDF or ABC/3TC] (*n =* 411)	87.8% *vs*. 85.4% (difference 2.5%; 95% CI –2.2 to 7.1).	Non-inferiority	[33]
	SINGLE	DTG + ABC/3TC (*n =* 414) *vs*. EFV/FTC/TDF (*n =* 419)	87.9% *vs*. 80.7% (difference 7%, 95% CI 2 to 12)	Non-inferiority with secondary superiority	[34]
	FLAMINGO	DTG + [FTC/TDF or ABC/3TC] (*n =* 242)* vs*. DRV + RTV + [FTC/TDF or ABC/3TC] (*n =* 242)	89.7% *vs*. 82.6% (difference 7.1%, 95% CI 0.9 to 13.2)	Non-inferiority with secondary superiority	[35]

RAL = raltegravir; BID = twice a day; FTC = emtricitabine; QD = once a day; EVG = elvitegravir; DTG = dolutegravir; CI = confidence interval; RTV = ritonavir; 3TC = lamivudine; TDF, tenofovir disoproxil fumarate; EFV, efavirenz; COBI, cobicistat; ATV, atazanavir; ABC, abacavir; DRV, darunavir.

Two phase 3 studies evaluated the EVG-based EVG/COBI/FTC/TDF regimen as a first-line therapy. In both studies, the primary endpoint was an HIV-1 RNA concentration of <50 copies/mL at Week 48 by intention-to-treat (according to the U.S. FDA snapshot algorithm), with a 12% non-inferiority margin. Study GS-US-236-0102 was a phase 3, randomized, double-blind study conducted in North America of EVG/COBI/FTC/TDF* vs*. EFV/FTC/TDF [31]. At Week 48, the proportion of patients with HIV-1 RNA <50 copies/mL was 88% (*n* = 305/348) in the EVG group compared to 84% (*n* = 296/352) in the EFV group (difference 3.6%, 95% CI −1.6% to 8.8%). Study GS-US-236-0103 was a phase 3, randomized, double-blind study conducted in Australia, Europe, North America and Thailand of EVG/COBI/FTC/TDF compared to ritonavir-boosted atazanavir (ATV + RTV) plus FTC/TDF [32]. At Week 48, the proportion of patients with HIV-1 RNA <50 copies/mL was 90% (*n* = 316/353) in the EVG group compared to 87% (*n* = 308/355) in the ATV + RTV group (difference 3.0%, 95% CI −1.9% to 7.8%). These EVG studies concluded the non-inferiority of EVG/COBI/FTC/TDF compared to EFV/FTC/TDF or ATV + RTV + FTC/TDF. 

Three phase 3 studies have been conducted with DTG in treatment-naive patients. In these studies, the primary endpoint was an HIV-1 RNA concentration of <50 copies/mL at Week 48 by intention-to-treat (according to the U.S. FDA snapshot algorithm) [33,34,35]. SPRING-2 was a phase 3, randomized, double-blind study in Canada, USA, Australia and Europe [33]. SPRING-2 compared DTG once daily to RAL twice daily, both in combination with either FTC/TDF or ABC/3TC. In this study, the proportion of patients with HIV-1 RNA <50 copies/mL at Week 48 was 88% (*n =* 361/411) in the DTG group, compared with 85% (*n =* 351/411) in the RAL group (difference 2.5%, 95% CI −2.2% to 7.1%), indicating that DTG was non-inferior to RAL [33]. SINGLE was a phase 3, randomized, double-blind study in North America, Europe and Australia [34]. SINGLE compared DTG once daily with ABC/3TC to EFV/FTC/TDF. At Week 48, the proportion of patients with HIV-1 RNA <50 copies/mL was 88% (*n =* 364/414) in the DTG group, compared with 81% (*n =* 338/419) in the EFV group (difference 7%, 95% CI 2% to 12%), indicating that DTG met the non-inferior margin and, in a pre-planned secondary analysis, was superior to EFV (*p* = 0.003), driven by differences in discontinuations due to adverse events [34]. FLAMINGO was an open-label, international, randomized study of DTG with FTC/TDF or ABC/3TC* vs*. ritonavir-boosted darunavir (DRV + RTV) plus FTC/TDF or ABC/3TC [35]. At Week 48, 90% (*n =* 217/242) of the DTG group achieved viral load <50 copies/mL* vs*. 83% (*n* = 200/242) of the DRV + RTV group (difference 7.1%, 95% CI 0.9% to 13.2%) and was non-inferior [35]. The pre-specified secondary analysis found DTG superior to DRV + RTV at Week 48 (*p* = 0.025), primarily driven by higher discontinuations in the DRV group [35].

### 2.4. Definitions of the Resistance Analysis Population and Resistance Testing Assays

Clinical trial protocols require formal descriptions of the criteria for the inclusion of a patient into the resistance analysis population (RAP). Guidelines require that plasma is collected and stored for later analyses of the development of resistance mutations. Generally, the specific samples to be analyzed are prospectively defined to adequately describe resistance development and provide for patient management. However, the choice of which sample(s) to study is not standardized between guidelines or between pharmaceutical companies. Within the development program of a drug, the resistance analyses may differ to fit the objectives of each particular study or phase of development. This is also an area of debate in routine clinical settings and is subject to budgetary limitations at hospitals, clinics, insurance companies and governments. 

In clinical trials and clinical practice, a variety of commercial and hospital-based assays are used for genotyping and phenotyping samples for HIV-1 drug resistance. Monogram Biosciences provided the resistance testing for IN in all of the trials reviewed here. The Monogram assay for PR and RT has been available for many years [36] and is now available for IN with the reporting of resistance to RAL, EVG and DTG. The Monogram assays are validated at viral loads >500 copies/mL according to the assay validation documents, but are routinely used for viral loads as low as 400 copies/mL. At lower viral loads, the success rate for the assay decreases and the resulting data may be less representative of the viral quasispecies due to PCR founder effects. 

The seven INSTI protocols utilized a variety of definitions for the resistance analysis population (Table 2). The RAP was similar for both STARTMRK and QDMRK and consisted of patients who had either non-response (suboptimal virologic response) or viral load rebound [29,30]. Non-response was defined as a patient who had ≥50 copies/mL of HIV-1 RNA at Week 24 or at early study discontinuation, without the achievement of <50 copies/mL at any stage of the study. Virologic rebound was two consecutive HIV-1 RNA measurements ≥50 copies/mL (>1 week apart) after an initial viral load response. Genotypic analysis of the IN, RT and PR genes were conducted on samples from patients included in the RAP who had HIV-1 RNA ≥400 copies/mL at the time of analysis, generally the first sample above 400 copies/mL in the case of a virologic rebound.

**Table 2:** Definitions of resistance analysis populations in INSTI clinical trials.

INSTI	Clinical Trial	Resistance Analysis Population	HIV-1 RNA Assay	Management of Patients with VF	Reference
RAL	STARTMRK and QDMRK	Non-response: ≥50 vRNA c/mL at Week 24 or ESD without achievement of <50 c/mL during the study. Rebound: after response, having two consecutive ≥50 vRNA c/mL (confirmed >1 week later). Resistance analysis on the first sample with HIV-1 RNA ≥400 c/mL.	COBAS Amplicor Monitor assay (v1.5)	Decision to discontinue by the Investigator	[29,30]
EVG	GS-US-236-0102 and GS-US-236-0103	Suboptimal virologic response: <1 log_10_ decrease in HIV-1 RNA from baseline by Week 8, confirmed at the next visit and ≥400 c/mL. Rebound: at any time after achieving <50 c/mL, two consecutive visits with ≥400 c/mL or two consecutive ≥1 log_10_ increases from nadir. Last Visit: at or after Week 8, HIV-1 RNA ≥400 copies/mL at the last visit (discontinuation for any reason or lost to follow up) or at Week 48, a single value of HIV-1 RNA ≥400 c/mL. The second, confirmation sample was tested, if available.	COBAS Amplicor Monitor assay (v1.5)	Continue study drug if no resistance detected, at the discretion of the Investigator	[31,32]
DTG	SPRING-2 and SINGLE	Between Week 24–48, two consecutive HIV-1 RNA values of ≥50 c/mL. First virologic failure sample analyzed.	RealTime HIV-1 PCR assay	Withdrawal from study required	[33,34]
	FLAMINGO	On or after Week 24, two consecutive HIV-1 RNA values of >200 c/mL. First virologic failure sample analyzed.	RealTime HIV-1 PCR assay	Withdrawal from study required	[35]

VF = virologic failure; RAL = raltegravir; EVG = elvitegravir; DTG = dolutegravir; ESD = early study discontinuation; c/mL = copies/mL; vRNA = HIV-1 viral RNA.

The definition of the RAP was identical in the EVG studies GS-US-236-0102 and GS-US-236-0103 [31,32]. All patients with virologic failure as defined below, or who had plasma HIV-1 RNA ≥400 copies/mL at Week 48, or at the time of early study drug discontinuation (at or after Week 8 and on study drugs) had samples tested for resistance. Virologic failure was defined as having either a suboptimal virologic response or virologic rebound. Suboptimal virologic response was an HIV-1 RNA <1 log_10_ decrease from baseline and ≥50 copies/mL by Week 8 and confirmed at the Week 12 visit. Virologic rebound was confirmed HIV-1 RNA ≥400 copies/mL after achieving <50 copies/mL or having two consecutive visits with ≥1 log_10_ increase in HIV 1 RNA from nadir. Virologic rebound required a confirmation unless the rebound occurred at Week 48, early study drug discontinuation or the last visit. The confirmed virologic failure sample, if available, was analyzed. Patients were allowed to continue in the study and on study drugs at the discretion of the investigator until emergent resistance was reported, and thus, additional later samples were tested in some cases.

The RAP was similar for both SPRING-2 and SINGLE and consisted of patients who had protocol-defined virologic failure between Week 24 and Week 48 [33,34]. Protocol-defined virologic failure was defined as two consecutive plasma HIV-1 RNA values of ≥50 copies/mL on or after Week 24. Patients meeting this criterion before Week 48 were withdrawn from the study unless study drug interruption was documented. Genotypic analyses of the IN, RT and PR genes was conducted on the first (suspected virologic failure) sample with HIV-1 RNA values of ≥50 copies/mL and was not restricted to viral load ≥400 copies/mL at the time of analysis. The FLAMINGO study utilized a similar definition of protocol-defined virologic failure as the previous two DTG studies, with the exception of a modification of the level of viremia to two consecutive plasma HIV-1 RNA values of >200 copies/mL at or after Week 24 [35]. In all of the DTG studies, potential virologic failure prior to Week 24 was not analyzed for resistance, as there were no criteria for virologic failure before the Week 24 time point in the study protocols.

There is a lack of standardization between approaches to study resistance development and how to manage patients with virologic failure. The similarities between these studies are that samples are collected and stored for analysis, and the assays used to study viral load and emergent drug resistance are similar. Differences that can lead to significantly discordant patient virologic outcome are the analysis of the first* vs*. the second virologic failure sample, the timeframe of inclusion in the resistance analysis population, the viral load criteria for resistance testing and protocol-specified discontinuation requirements.

### 2.5. Development of Resistance in Clinical Trials of INSTIs

In most cases during the development of a drug, resistance analyses consist of genotyping (sequencing) the HIV-1 viral gene coding regions targeted by the drug and phenotyping against the drug and a panel of other inhibitors. In the cases of the INSTI studies described here, resistance and cross-resistance to the INSTIs that were approved at the time of study were evaluated. The specific sample analyzed, such as the first above a viral load threshold, the confirmatory sample and/or subsequent samples, differs between studies and within clinical practice. Most analyses in these clinical trials utilized similar assays for virologic failure sample analysis. Through Week 48, emergent resistance to the INSTI, other class and NRTI backbone is described (Table 3). In the studies reviewed here, resistance to a study drug was infrequent (<3% of treated patients) [29,30,31,32]. Resistance was more frequent in the experimental QD RAL arm of the QDMRK study at 5.2% of patients [30]. In the DTG treatment groups of the treatment-naive studies, no resistance has been reported [33,34,35]. For studies with a longer follow-up, the rate of resistance decreases as the time of suppression increases [37,38,39,40,41]. 

**Table 3:** Resistance development in INSTI clinical trials.

INSTI	Clinical Trial	Treatment Group	Resistance Analysis Population; n (%) ^a^	Emergent INSTI Resistance; n (%)	Emergent Other Resistance; n (%)	Reference
RAL	STARTMRK	RAL (BID) + FTC/TDF	9 (3.2%)	4 (1.4%)	3 (1.1%)	[29]
		EFV + FTC/TDF	7 (2.5%)	na	3 (1.1%)	
	QDMRK	RAL (QD) + FTC/TDF	30 (7.9%)	9 (2.4%)	20 (5.2%)	[30]
		RAL (BID) + FTC/TDF	16 (4.1%)	2 (0.5%)	6 (1.5%)	
EVG	GS-US-236-0102	EVG/COBI/FTC/TDF	14 (4.0%)	7 (2.0%)	8 (2.3%)	[31]
		EFV/FTC/TDF	17 (4.8%)	na	8 (2.3%)	
	GS-US-236-0103	EVG/COBI/FTC/TDF	12 (3.4%)	4 (1.1%)	4 (1.1%)	[32]
		ATV + RTV + FTC/TDF	8 (2.3%)	0	0	
DTG	SPRING-2	DTG + [FTC/TDF or ABC/3TC]	20 (4.9%)	0	0	[33]
		RAL + [FTC/TDF or ABC/3TC]	28 (6.8%)	1 (0.2%)	4 (1.0%)	
	SINGLE	DTG + ABC/3TC	18 (4.3%)	0	0	[34]
		EFV/FTC/TDF	17 (4.1%)	na	4 (1.0%)	
	FLAMINGO	DTG + [FTC/TDF or ABC/3TC]	2 (0.8%)	0	0	[35]
		DRV + RTV + [FTC/TDF or ABC/3TC]	2 (0.8%)	0	0	

RAL = raltegravir; EVG = elvitegravir; DTG = dolutegravir; na = not applicable; ^a^ % is the n divided by the number of treated patients.

Resistance in the RAL studies was infrequent and often consisted of both INSTI and NRTI resistance mutations. Through Week 48 of STARTMRK, the HIV-1 from nine patients was analyzed for resistance development in the RAL group. Of these, eight of nine samples had genotypic information available for IN, and four patients had emergent INSTI resistance mutations (three also had data for RT, and all had FTC resistance mutations) [29]. In the QDMRK study, the resistance analysis population and those with emergent resistance were higher in the QD RAL group compared to the BID RAL group [30]. In the QD RAL group, 30 patients (7.9%) were included in the RAP, and of those, nine had INSTI-R (2.4%) and 20 (5.2%) had NRTI-R. In the BID RAL group, 16 (4.1%) of the patients were included in the RAP; two (0.5%) had INSTI-R, and six (1.5%) had NRTI-R. In both studies, INSTI-R primarily consisted of N155H, Y143C/R, Q148H/R/K or E92Q, consistent with earlier studies of RAL in treatment-experienced patients [15]. Overall, across both studies, all patients with data available for both genes who had INSTI-R also had NRTI-R. 

Resistance in the EVG studies was also infrequent and consisted of INSTI-R and NRTI-R in most cases. Through Week 48 in Study GS-US-236-0102, there were 14 patients analyzed for resistance in the EVG/COBI/FTC/TDF group, and all had data available for RT and IN. Eight patients (2.3% of the randomized population) had emergent resistance to a study drug [31]. Seven patients had an INSTI resistance mutation (2.0%), and eight had an NRTI resistance mutation (2.3%). In Study GS-US-236-0103, there were 12 patients analyzed for the development of resistance in the EVG/COBI/FTC/TDF group [32]; 10 had data available for both RT and IN genes, and two had data only for IN. Five patients had NRTI and/or INSTI emergent resistance. One patient had only M184V in RT; one patient had HIV-1 with INSTI-R and no data for RT, and three had INSTI-R in combination with NRTI-R. Overall, the primary INSTI-R mutations for EVG were predominantly E92Q, followed by Q148R, N155H and T66I. These INSTI-R mutations were similar to those observed in earlier studies of EVG in treatment-experienced patients [42]. Similar to the pattern of resistance in the RAL studies, all cases of INSTI-R with data for RT also had NRTI-R.

The DTG treatment-naive patient studies reported no emergent resistance in any of the INSTI-containing arms of the studies. Through Week 48 in the SPRING-2 study, the HIV-1 from 20 patients was analyzed for resistance development in the DTG group and from 28 patients in the RAL group [33]. The resulting data was limited to eight patients for IN and 12 patients for RT in the DTG group, and no resistance to study drugs was found in any of these. In the RAL group, there were IN results for 18 patients and RT results for 19 patients, with one patient having emergent resistance to RAL, FTC and TDF and three patients having an NRTI-R mutation. In the SINGLE study, 18 patients in the DTG group had virologic failure and resistance testing attempted [34]. The number of patients with available data was limited due to the low viral load (<200 copies/mL) at the time of the first failure in 16 of 18 patients. In the final analysis, IN and RT data was available for seven patients and nine patients, respectively, and no patient had emergent resistance to study drugs. In FLAMINGO, two patients in the DTG group and two patients in the DRV + RTV had virologic failure and were analyzed for resistance, with no resistance mutations detected in either group [35]. Overall, there were no emergent resistance mutations detected in the DTG groups in these first-line studies. In the treatment-experienced, but INSTI-naive, patients, virologic failure on DTG-containing regimens had four cases with emergent DTG-R mutations (R263K, *n =* 2; V151I, *n =* 1; E138T/A + T97A with preexisting Q148H, *n =* 1) [43]. Given these limited data, the* in vivo* resistance pathways for DTG in treatment-naive patients will require further study and might be observed in patients who are maintained for more extended periods on a failing regimen. In the clinical studies of DTG, treatment-naive patients were rapidly discontinued from study per protocol upon confirmation of >50 copies/mL of HIV-1 RNA, which prevented extended virologic failure on the treatment.

## 3. Clinical Virology Case Studies

**Figure 1:**
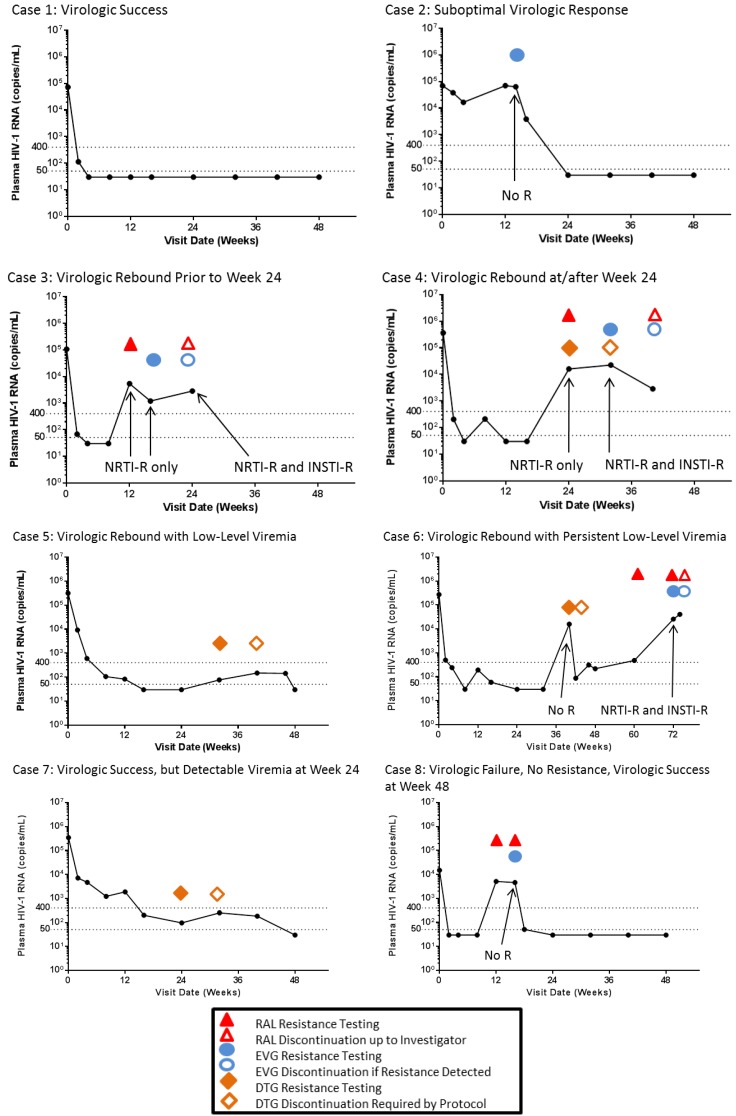
Resistance analyses and HIV-1 RNA profiles of eight clinical case studies by study visit. Plasma HIV-1 RNA in copies/mL are indicated on the y-axis, with values at 50 copies/mL and 400 copies/mL indicated by the dotted horizontal lines. The time in weeks of the scheduled and unscheduled study visits are indicated on the x-axis. The viral load at each visit is plotted (black circles and black lines). Emergent resistance to the NRTI or INSTI class is indicated at specific visits with an arrow and text. The RAL Protocols use red triangles (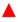), the EVG Protocols blue circles (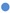) and the DTG Protocols orange diamonds (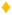). Filled symbols represent the point at which resistance testing would be conducted. Open symbols represent the visit where a patient would be discontinued from study drugs. No R indicates no nucleos(t)ide reverse transcriptase inhibitor (NRTI) or INSTI resistance emerged.

To illustrate some of the differences between inclusion in the resistance analysis population and patient management by the different protocols of INSTI trials in treatment-naive patients, clinical cases from the GS-US-236-0102 and GS-US-236-0103 studies are presented and discussed (Figure 1). Most cases present plasma HIV-1 viral loads from baseline and through either the last observation point at study drug discontinuation or through Week 48. For brevity, the STARTMRK and QDMRK studies will be referred to as the “RAL protocols”; Studies GS-US-236-0102 and GS-US-236-0103 will be referred to as the “EVG protocols”, and SPRING-2, SINGLE and FLAMINGO will be referred to as the “DTG protocols”. In the DTG studies, when there is a difference between SPRING-2 SINGLE and FLAMINGO, it will be noted as such. Some of these cases are straight-forward, and others are more difficult to retrospectively characterize as to how they would have been managed according to the different protocols. The clinical management of patients described in the RAL and DTG protocols has been imputed based on publications describing these protocols.

### 3.1. Case 1: Virologic Success 

Case 1 is a typical example of the rapid virologic suppression achieved by an INSTI-based regimen (Figure 1). This is a 24-year-old male with a baseline HIV-1 RNA of 72,300 copies/mL who was randomized and treated with EVG/COBI/FTC/TDF, had a rapid decrease in viral load to 113 copies/mL at Week 2 and achieved undetectable viral load (<50 copies/mL) at all visits from Week 4 through Week 48. As described in the efficacy and results section, rapid suppression and maintenance of HIV-1 RNA <50 copies/mL through Week 48 was achieved in 83% to 90% of INSTI-treated patients spanning the seven trials. These patients would be considered virologic successes by all seven protocols with no indication for resistance testing conducted post-baseline.

### 3.2. Case 2: Suboptimal Virologic Response

Case 2 is an example of a patient with initial suboptimal virologic response followed by virologic suppression. This is a 54-year-old male with a baseline HIV-1 RNA of 69,500 copies/mL, who was randomized and treated with EVG/COBI/FTC/TDF and had a weak virologic response of −0.62 log_10_ copies/mL by Week 4. At Week 4, the patient was given a 30-day supply of study drugs. He missed his Week 8 visit, but returned for his Week 12 visit and had a viral load comparable to baseline (70,000 copies/mL), consistent with non-adherence, due to running out of study medication. At the Week 12 visit, the patient in Case 2 was given another 30-day supply of study drugs, but upon a Week 12 retest visit 11 days later, the viral load was still high at 63,400 copies/mL. Subsequently, the viral load decreased to 3820 at Week 16 and was <50 copies/mL at Week 24 through Week 48.

The patient in Case 2 had a suboptimal virologic response by the EVG protocols, and the suboptimal virologic response failure confirmation sample (Week 12 retest) was sent for genotypic and phenotypic resistance testing. No resistance mutations were found in the HIV-1 RNA from the Week 12 retest sample. EVG/COBI/FTC/TDF was continued successfully through Week 48. According to the RAL and DTG protocols, because the viral load was <50 copies/mL at Week 24, no sample would have been analyzed for resistance. In the RAL and DTG protocols, Case 2 would be considered a virologic success, while in the EVG protocols, he was counted in the resistance analysis population, but was a virologic success at Week 48.

### 3.3. Case 3: Virologic Rebound Prior to Week 24

Case 3 is an example of a patient with viral load suppression followed by a virologic rebound to ≥400 copies/mL that was confirmed at the next visit. Virologic rebound and confirmation of rebound occurred prior to Week 24. This is a 37-year-old female with a baseline HIV-1 RNA of 107,000 copies/mL, who was randomized and treated with EVG/COBI/FTC/TDF and had an initial viral load response of <50 copies/mL achieved at Week 4 and confirmed at Week 8. At Week 12, the viral load was found to be 5410 copies/mL, which remained elevated at Week 16 (1190 copies/mL) and at Week 24 (2840 copies/mL). 

Case 3 was considered a virologic rebound by the EVG protocols and had the failure confirmation sample (Week 16) sent for genotypic and phenotypic resistance testing. By the RAL protocols, virologic rebound to >400 copies/mL was confirmed, and the Week 12 sample would have been tested. If this showed no mutations, then the Week 16 sample would also have been sent for resistance testing. If resistance was found, then this patient would have been discontinued at a later visit, at the discretion of the Investigator. According to the DTG protocols, the rebound occurred prior to Week 24, and no sample would have been sent for resistance testing, unless the rebound was confirmed on or after the Week 24 visit window. The DTG protocols required a confirmation visit within 1–4 weeks after the suspected virologic failure. In this case, this next visit would have confirmed the virologic failure, and the Week 24 sample would have been sent for resistance testing. It is also possible that the patient in Case 3 could have been discontinued prior to Week 24, in which case, no sample would be analyzed for the emergence of resistance (as shown in Figure 1). 

At screening, the patient in Case 3 had HIV-1 with V118I in RT and no mutation in IN when retrospectively tested at baseline. At Week 16, the K65K/R and M184M/I resistance mutations in RT were detected, and the patient was discontinued due to lack of efficacy at Week 24 once these results were obtained. At Week 16, no resistance was found in the IN gene; however, multiple genotyping attempts were required to obtain this result. *Post hoc* analysis of the first failure Week 12 sample reported only the M184M/I mutation in RT and no mutation in IN. An additional *post hoc* analysis of the discontinuation visit sample from Week 24 by clonal sequencing found clones with a dominant resistance pattern of V118I + M184I in RT and E92Q in IN [44]. Case 3 is a good example of the accumulation and evolution of resistance patterns in patients who remain on a failing regimen. This case represents one of the cases where virologic failure and resistance occurred prior to Week 24.

### 3.4. Case 4: Virologic Rebound at/after Week 24

Case 4 is an example of a patient with viral load suppression followed by a virologic rebound to ≥400 copies/mL that was confirmed at the next visit. This is a 38-year-old male with a baseline HIV-1 RNA of 364,000 copies/mL, who was randomized and treated with EVG/COBI/FTC/TDF. This patient had an initial viral load response with HIV-1 RNA <50 copies/mL first achieved at Week 4, followed by transient viremia of 209 copies/mL at Week 8 and resuppression to <50 copies/mL at Week 12. At Week 24, the viral load rebounded to 15,900 copies/mL and increased to 22,400 copies/mL at Week 32.

By all seven protocols, Case 4 would be considered a virologic failure/rebound. The RAL and DTG protocols would have studied the first sample with >400 copies/mL once a confirmation visit had occurred. In contrast, the EVG protocols analyzed the failure confirmation sample (Week 32) for emergent resistance. 

Resistance mutations in RT and IN were detected in the Week 32 sample (M184V in RT and E92E/Q, Q148Q/R and N155N/H in IN), and the patient was discontinued due to lack of efficacy at the Week 40 visit once the results were obtained. *Post hoc* analysis of the first failure Week 24 sample found M184V in RT and no mutation in IN. An additional *post hoc* analysis of the discontinuation visit sample from Week 40 by clonal sequencing found clones with four main resistance patterns: M184V + Q148R, M184V + E92Q, M184V + N155H, and M184V alone [44]. Case 4 is also a good example of the accumulation and rapid evolution of resistance patterns in patients who remain on a failing regimen and that it is rare for primary INSTI-R mutations to be linked on the same genome [45]. 

Patient management would have been different by these protocols. By the EVG protocols, the patient from Case 4 was to be discontinued as soon as the resistance results showing resistance mutations to a study drug were obtained, as was done in this case. By the RAL and DTG protocols, Case 4 would have been brought back in for a viral load retest (within 1–4 weeks after the Week 24 suspected virologic failure visit for the DTG protocols) and discontinued if the confirmation result was >50 copies/mL for SPRING-2 and SINGLE and if >200 copies/mL for FLAMINGO, which was satisfied by the data available at Week 32. The decision to discontinue the patient was made by the investigator for the RAL protocols, but likely would have been as soon as resistance was detected. Based on population sequencing data and more rigorous retest/return visits required by the RAL and DTG protocols, the development of resistance mutation in the IN gene may have been prevented. 

### 3.5. Case 5: Virologic Rebound with Low-Level Viremia

Case 5 is an example of a patient with viral load suppression followed by an extended period of low-level viremia with virologic success of HIV-1 RNA <50 copies/mL at Week 48. This is a 42-year-old male with a baseline HIV-1 RNA of 315,000 copies/mL, who was randomized and treated with EVG/COBI/FTC/TDF. He had an initial viral load response, and a viral load of <50 copies/mL was first achieved at Week 16 and was confirmed at Week 24. At Week 32, a low-level viremia of 77 copies/mL was first detected, and the low-level viremia continued through Week 48. After Week 48, a retest was performed and showed HIV-1 RNA <50 copies/mL. 

By the DTG protocols of SPRING-2 and SINGLE, Case 5 would be a virologic rebounder. The suspected virologic failure sample (first failure sample; Week 24) would have been analyzed, and this patient would have been discontinued at the next visit. The FLAMINGO protocol required rebound viremia to exceed 200 copies/mL, and therefore, Case 5 would not be considered a virologic rebound. The RAL protocols would not have categorized Case 5 as a having had a rebound, because the last value in the Week 48 window was <50 copies/mL. By the EVG protocols, Case 5 was not considered to have a rebound, because the viral load was not ≥400 copies/mL at two consecutive visits. The patient in Case 5 continued their blinded EVG/COBI/FTC/TDF treatment and had HIV-1 RNA <50 copies/mL at all subsequent study visits from Week 48 through Week 144.

### 3.6. Case 6: Virologic Rebound with Persistent Viremia

Case 6 is an example of a patient with viral load suppression followed by a virologic rebound. This was a 42-year-old male with a baseline HIV-1 RNA of 273,000 copies/mL, who was randomized and treated with EVG/COBI/FTC/TDF. This patient had an initial viral load response, and a viral load of <50 copies/mL was first achieved at Week 8, followed by transient low-level viremia and resuppression at Week 24. At Week 40, there was a viral load rebound to 15,800 copies/mL that was followed by persistent, low-level viremia and HIV-1 RNA >400 copies/mL at Week 60 that was confirmed at Week 72.

By all protocols, Case 6 would be considered a virologic rebound; however, the time at which the failure was confirmed and tested for emergent resistance would vary by protocol. The DTG protocols would have led to the earlier testing of Case 6 at Week 40 for SINGLE and SPRING-2 after two consecutive visits with >50 copies/mL. The Week 48 sample would have had resistance testing by FLAMINGO, being the first failure sample of two consecutive visits with >200 copies/mL. The RAL and EVG protocols required a higher viral load for testing. The RAL protocols would have analyzed the first sample with >400 copies/mL at Week 60, and the EVG protocols analyzed the confirmation sample with viral load ≥400 copies/mL at Week 72. 

For Case 6, resistance mutations in RT and IN were detected in the Week 72 sample (M184V in RT and E92Q in IN), and the patient was discontinued due to lack of efficacy at the Week 72 retest visit once the results were obtained. *Post hoc* analysis of the first failure Week 40 sample found no mutations in RT or IN. Case 6 is a good example of the most frequently occurring pathway of resistance to EVG/COBI/FTC/TDF of M184V/I + E92Q and is similar to the combination resistance of M184V/I plus INSTI-R in RAL + FTC/TDF virologic failures [29,30,31,32]. 

Patient management would have been different by these protocols. By the EVG and likely the RAL protocols, the patient in Case 6 was to be discontinued as soon as the resistance results finding resistance to study drugs were obtained, as was done for this patient. By the DTG protocols, this patient would have been brought back in for a viral load retest within 1–4 weeks after the Week 40 suspected virologic failure, which did occur, and discontinued if the result was >50 copies/mL for SPRING-2 and SINGLE (Week 40 retest) and if >200 copies/mL for FLAMINGO (Week 48 retest). Based on population sequencing of the Week 40 sample, the emergence of resistance was not detected. The requirement for a higher viral load at virologic failure may have led to the longer treatment of this patient on a failing regimen, and the earlier change in therapy may have limited the emergence of resistance mutations in both RT and IN. However, several cases of confirmed rebound and no emergent resistance followed by sustained suppression were also observed, as will be described in Case 8.

### 3.7. Case 7: Virologic Success, but Detectable Viremia at Week 24

Case 7 is an example of a patient with slow viral load suppression that took until Week 48 to reach a viral load of <50 copies/mL. This is a 52-year-old female with a baseline HIV-1 RNA of 346,000 copies/mL, who was randomized and treated with EFV/FTC/TDF, had an initial viral load response greater than 1 log_10_ copies/mL by Week 2. At Week 24, the viral load was 96 copies/mL. Low-level viremia was reported at Weeks 32 and 40 of 252 copies/mL and 185 copies/mL, respectively. At Week 48, the viral load was <50 copies/mL for the first time.

Case 7 would have been managed differently by the INSTI clinical protocols. Since the viral load was <400 copies/mL and was undetectable at Week 48, the RAL protocols would not call for resistance testing or patient discontinuation. Because the virus had >1 log_10_ decrease in viral load by Week 8 and did not have a rebound to >1 log_10_ from nadir, or two consecutive ≥400 copies/mL viral loads, the EVG protocols would also not call for resistance testing or patient discontinuation. The FLAMINGO protocol would not call for resistance testing, because the viral load was not >200 copies/mL at two consecutive visits. According to the DTG protocols (with the exception of FLAMINGO), since the HIV-1 RNA was >50 copies/mL at Week 24 and confirmed at the next visit, a resistance test would have been conducted on the Week 24 sample, and the patient in Case 7 would be discontinued after the Week 32 visit (or earlier if a retest was done 1–4 weeks after Week 24 in this case, as required). Since this patient was suppressed at Week 48, there would have likely been no resistance, and this patient would have been discontinued and considered a virologic failure, while in the FLAMINGO, as well as in the EVG and RAL protocols, this patient was considered as a virologic success.

### 3.8. Case 8: Virologic Failure, No Resistance, Virologic Success at Week 48

Case 8 is an example of a patient with viral load suppression followed by confirmed virologic rebound to viral load ≥400 copies/mL, no evidence of emergent resistance, followed by resuppression and virologic success at Week 48. This is a 22-year-old female with a baseline HIV-1 RNA of 14,900 copies/mL who was randomized and treated with EVG/COBI/FTC/TDF. A viral load <50 copies/mL was first achieved at Week 2 and maintained through Week 8. At Week 12, the viral load was 5080 copies/mL, and the viral load rebound was confirmed at Week 16, with 4600 copies/mL. At Week 16, a retest measured the viral load at 51 copies/mL, followed by <50 copies/mL at all subsequent visits through Week 48. 

Case 8 would be managed differently by the INSTI protocols. The EVG studies considered Case 8 a virologic rebound and sent the failure confirmation sample (Week 16) for genotypic and phenotypic resistance testing. No resistance to study drugs was found, and the patient continued on study drugs and was considered a virologic success at Week 48. According to the RAL and DTG protocols, the rebound occurred prior to Week 24 (DTG protocols) or the patient resuppressed (RAL protocols), and no sample would have been analyzed for resistance. 

## 4. Discussion 

Current initial treatments for HIV-1 infection are highly effective, have good safety profiles and are generally well tolerated. As antiretroviral drugs have improved in potency and tolerability, combined with the greater simplicity of the newest combinations, such as the single-tablet regimens, adherence is facilitated and the frequency of virologic failure and emergent resistance has declined. Nevertheless, emergent resistance can occur, which can compromise the activity of ongoing regimens under the selective pressure of persistent viremia and limit the remaining choices for future therapy. Thus, characterization of the resistance profile of a new drug through clinical development continues to be critical. Moreover, the development of new classes of antiretrovirals with high resistance barriers that do not overlap with other drug classes is still needed. In addition, with the high rate of efficacy currently achieved of up to 90% and more sensitive assays for the detection of low-level viremia and minority resistance variants, the choice of study design and the resistance analysis plan is important, so that the best information on future clinical care is obtained.

There remain many questions regarding resistance analyses and virologic failure, such as which genes to sequence prior to therapy, which viral load signifies virologic failure, which samples to test for resistance and when to change a failing regimen. Furthermore, standards will vary among clinical trials, clinical practice and by region. Resistance testing at failure for the EVG studies was performed on confirmed virologic failure samples with HIV-1 RNA ≥400 copies/mL. For the RAL studies, testing was performed at the first failure with HIV-1 RNA >400 copies/mL, while in SPRING-2 and SINGLE, genotyping was performed on the first sample from virological failures at or after Week 24 with HIV-1 RNA >50 copies/mL. Such differences between study protocols limit cross-study comparison, as the resistance data generated depends on the resistance analysis time point, which may allow for periods of low or intermediate viral replication prior to analysis. Indeed, conducting a genotypic test on the first sample above 50 copies/mL does not provide the same information as a genotype confirmatory sample ≥400 copies/mL, the latter situation being the one most often followed in clinical practice.

In the seven phase 3 clinical trials of INSTI-based regimens as a first-line therapy presented here, high efficacy outcomes were observed in the absence of pre-treatment genotypic information for the IN gene [29,30,31,32,33,34,35]. Eventually though, transmission of INSTI-R may reach levels high enough to recommend including IN genotyping prior to therapy. 

The definition of what viral load constitutes virologic failure also continues to be debated and will likely depend on the regimen. Intense discussion on patient management in the setting of low-level viremia and the risk of virologic failure are also ongoing [46,47]. Virologic failure at low levels (e.g., between 40–200 copies/mL), where resistance data may not be available, may be cause for some clinicians to make a regimen change to prevent greater levels of viral replication and possibly resistance development [46,48]. 

In this review, seven major randomized and controlled clinical trials of INSTI-based initial treatment of HIV-1 infection showed high efficacy outcomes. The rate of resistance remains low for first generation INSTIs and has, to date, been absent for DTG in treatment-naive patients. However, differences in clinical protocols and resistance testing may have contributed to some of the differences in the study and resistance outcomes among these trials. These differences underscore the challenges in comparing the resistance profiles of new drugs characterized in different clinical development programs. Greater consistency in approaches to resistance analyses will help; however, evolving clinical standards and assays will likely hinder universal conformity. The INSTI class has been the most recently introduced class of antiretroviral drugs and has shown strong efficacy and safety. INSTI-based regimens are now providing for another highly-effective therapeutic option for HIV-1-infected patients. 
